# The Benefits of Combining Bobath and Vojta Therapies in Infants with Motor Development Impairment—A Pilot Study

**DOI:** 10.3390/medicina59101883

**Published:** 2023-10-23

**Authors:** Daniela Parau, Anamaria Butila Todoran, Laura Barcutean, Calin Avram, Rodica Balasa

**Affiliations:** 1 Doctoral School, ‘George Emil Palade’ University of Medicine, Pharmacy, Science, and Technology of Targu Mures, 540142 Targu Mures, Romania; daniela.parau@yahoo.com; 2 Department of Genetics, ‘George Emil Palade’ University of Medicine, Pharmacy, Science, and Technology of Targu Mures, 540142 Targu Mures, Romania; 3 Department of Neurology, ‘George Emil Palade’ University of Medicine, Pharmacy, Science, and Technology of Targu Mures, 540136 Targu Mures, Romania; rodica.balasa@umfst.ro; 4 Department of Medical and Biostatistics Informatics, ‘George Emil Palade’ University of Medicine, Pharmacy, Science, and Technology of Targu Mures, 540136 Targu Mures, Romania; calin.avram@umfst.ro

**Keywords:** Bobath, Vojta, kinesiotherapy, motor recovery, infants

## Abstract

*Background*: In infants presenting with motor development impairment, early kinesiotherapeutic interventions aim to normalise the pattern of movements and improve recovery. By applying Bobath and Vojta methods, we aimed to identify a combined approach regarding motor deficit in infants with neurological disabilities. *Methods*: We designed a prospective interventional study on 108 infants with motor developmental delay and applied Bobath, Vojta, or combined Bobath and Vojta therapy in three equal groups. *Results*: In the combined Bobath and Vojta group, complete motor recovery was achieved for 50% of the participants, with full recovery after six months, whereas in Bobath- or Vojta-only therapy groups, the total recovery for all participants was achieved at seven months. Regarding infants with muscular hypertonia, Bobath therapy initiation demonstrated complete recovery in 5 months in more than 50% of the cases, while for Vojta this was achieved in only 33.57% of the cases. *Conclusions*: The comparative evaluation conducted by analysing the data regarding the application of the Bobath and Vojta methods showed that combining these two therapies results in a shorter motor deficit recovery time than if a single therapy is applied. These findings have important implications for the selection of rehabilitation therapies in infants with neurological motor development issues.

## 1. Introduction

Kinesiotherapy represents a therapeutic intervention aiming to normalise the pattern of movement. The principle of kinesiotherapy is based on a series of inhibitory and facilitatory stimuli. The external stimuli carry an ascending pattern of muscular or cutaneous origin, and the central stimuli have a descending pattern with a conscious, motivational, and emotional component [1]. Neuro-motor recovery techniques are based on inhibitory and facilitatory processes that produce voluntary muscle control and global movement. The array of stimuli with an exteroceptive, proprioceptive, labyrinth, or vestibular nature educates and shapes automatic motricity, the fundament of active voluntary movement [2]. In the process of neuro recovery for children with cerebral motor disabilities, the kinesiotherapeutic treatment considers the motor development–adjusted age and not the chronologic age. Therefore, a thorough knowledge of neuro-motor milestone development is essential. Motor deficit recovery is based on the concept of neuroplasticity, the brain’s ability to form and model new synapses [2,3]. Brain lesions result in a disorganised and delayed development of postural, equilibrium, and movement neurological mechanisms. The muscles required for these motor skills generate inefficient and uncoordinated movements, such as increased muscle tone (hypertonia) and decreased muscle tone (hypotonia). In addition to neuromuscular components, motor dysfunction has musculoskeletal involvement, with orthopaedic spine deformities (kyphosis, scoliosis) [2,4].

Motor development issues in infants can have long-term effects on their quality of life and independence. Motor developmental issues manifest in impaired motor function and affect approximately 3–4% of infants [2,5]. If they are identified in time, early therapeutic intervention can improve or even eliminate the dysfunctions, and thus, age-corresponding clinical parameters can be obtained [6]. A wide range of antenatal, perinatal, and postnatal factors contribute to the risk of brain injury. Among the most common antenatal risk factors are infections with herpes, rubella, toxoplasma, and cytomegalovirus, which can lead to irreversible brain lesions, Rh incompatibility, metabolic and genetic anomalies, hypoglycaemia, arterial hypertension, and pelvic/abdominal trauma of the mother [7,8,9]. Perinatal risk factors encompass the gestational age, in utero foetal position, obstetrical interventions, weight, APGAR score, hypoxia and the need for oxygen therapy after delivery, epileptic seizures, intracranial haemorrhage, and central nervous system (CNS) infections [10,11,12,13]. Post-natal risk factors include all the events occurring after the seventh day following delivery until two years of age, such as systemic disorders, head trauma, CNS infections, metabolic disorders (hypocalcaemia, hypoglycaemia, and hypoxia/anoxia secondary to epileptic seizures), epileptic seizures, and ischemic or haemorrhagic stroke [14,15,16,17,18].

According to Vaughan-Graham et al., the clinical rationale behind the kinesiotherapeutic intervention is based on integrating postural control and selective movements to optimise recovery using the Bobath method [19,20]. The neurodevelopmental therapy of the Bobath method represents a positioning system aiming to diminish or eliminate primitive tonic reflexes and to develop conditional, recovery, stretching, and body orientation reflexes in relation to gravity. Kinetic therapy aims to stimulate and teach the acquisition of appropriate motor skills to correct vicious postures and uncoordinated movements so that the child adapts to the environment as much as possible by forming the necessary functional abilities [21,22,23]. Vojta therapy, or the reflex locomotion method, targets specific reflex points and well-defined body postures that trigger motor involuntary and reciprocal reactions on the trunk and extremities [24]. These motor patterns have the characteristics of locomotion. The Vojta method was developed by Czech neurologist Vaclav Vojta, who assumed that cerebral palsy should be considered a functional blockage in the movement development process. This method evokes target-oriented coordination in the face of spontaneous movement, resulting in uprighting the body against gravity and augmenting grasping function and speech [25].

This study aimed to develop a new approach regarding motor deficit recovery in infants and children with neurological disabilities resulting from ante-, peri- and post-natal complications. Our research question is whether combining Vojta and Bobath therapies yields more favourable outcomes in infant neurorehabilitation than using each therapy alone. We hypothesise that the implementation of explicit criteria for the different types of procedures, with a well-defined sequence, frequency, intensity, and duration, will have beneficial effects on the neuromuscular system through the formation of motor en-grams that facilitate new patterns of movement and achieve a level of motor development as close as possible to physiological objectives.

## 2. Materials and Methods

### 2.1. Study Design

We designed a prospective interventional study to be carried out over two years, 2020–2022, in Mures county, Romania, that included infants with ante-, peri-, and post-natal complications and diagnosed with neuro-reflex-motor developmental delay. 

The study was approved by the Scientific Ethics Committee of the University of Medicine, Pharmacy, Science and Technology “George Emil Palade” of Targu Mures, No. 926/03.06.2020. Written, informed consent was obtained from all caregivers before study participation.

### 2.2. Participants

We included 108 infant patients with neuro-reflex-motor developmental delay, grouped by muscular characteristics: hypotonic, spastic, and mixed. The subjects were assessed based on clinical and demographical criteria and the leading cause of neuromotor developmental delay. Patients were randomly distributed into three equal groups, to which Bobath and Vojta therapies were applied: group one—Bobath therapy; group two—Vojta therapy; group three—both Bobath and Vojta combined in the same session.

The inclusion of patients in our study was determined through neurological consultations. All patients were diagnosed with delayed motor development, with one of the contributing factors being hypoxia. The diagnosis was made according to the aetiology of the triggering factors of hypoxia (ante-, peri-, post-natal); at the same time, using archaic reflexes [26], changes in muscle tone were highlighted, with the neurological condition being grouped in the hypotonic, spastic, and mixed forms.

To determine the level of motor development, a kinesiotherapeutic evaluation was carried out based on the motor functional evaluation scale—the ideal motor development scale of the child, according to Dr. Vojta V. (2001). This was supplemented by specialty medical evaluations: orthopaedic and neurosurgical, as applicable.

The inclusion criteria were (1) infants between 0 and 6 months of age with the neurological diagnosis of neuromotor developmental delay; (2) signed, informed consent from the caregivers for study participation. 

The exclusion criteria were (1) patients with acute or chronic psychological disturbances; (2) CNS infections (meningitis, encephalitis); (3) non-compliance; (4) refusal to sign the informed consent forms for study participation. The patient assessment was made according to the Bayley scale for Infant and Toddler Development and by the Vojta principles [24,27]. 

The subjects were assessed based on clinical and demographic criteria, as well as the primary cause of neuromotor developmental delay. Three equal groups were formed, to which Bobath and Vojta therapies were applied: group one received Bobath therapy, group two received Vojta therapy, group three received both Bobath and Vojta therapies, combined in the same session. These groups were homogeneous, as all three consisted of subjects of the same age, with consideration of months since birth, the same average birth weight, and the same neuro-reflex-motor developmental delay. 

The inclusion of patients in our study was based on the results of neurological consultations, considering motor development delays primarily attributed to ante-, peri-, and postnatal factors, with hypoxia being a significant manifestation in the latter two. Furthermore, we evaluated infants with delayed motor development and associated mild degrees of hypoxia, who were referred for outpatient rehabilitation treatment. All subjects came from similar socio-economic and cultural backgrounds.

### 2.3. Procedures 

The chronological infant age was confounded against the neuro-motor developmental age. The two methods were applied to all study participants, by a specialised kinesio-therapist certified in Vojta therapy, over a period of seven months in total, and motor recovery was noted. The therapy sessions were carried out in the presence of the carers; on occasion, they were informed about certain simple procedures that they could perform at home.

In order to establish the stage of motor deficiency, the infants were positioned in dorsal and ventral decubitus, and the spontaneous motility of the whole body as well as movement sequences was evaluated, thus being placed at a certain level of development (corresponding to the age of 0–2 years), a level that was then compared with the ideal motor development ontogeny (V. Vojta 2001).

The ***Vojta therapy*** programme was based on the following criteria:

1. The reflex roll from the supine position—Phase I, from side decubitus; phase II, III, and IV, with reflex activation of two or three points.
2. Reflex crawling from the prone position with reflex activation of two or three points and the first position (crouching at the edge of the bed).

The duration of the 30 min session was started by positioning the infant on the therapy table in 3 positions: dorsal decubitus (DD), ventral decubitus (DV), and lateral decubitus (DL). The activation time was 5 min for each position, with a 5 min break between positions.

The ***Bobath therapy*** was applied based on the following criteria:

1. Righting reactions—maintaining the normal position of the head in space and normal alignment with the trunk, and the trunk with the upper and lower limbs.
2. Balancing reactions—the visual, vestibular, and proprioceptive pathways are trained through posture changes.
3. Verticalization reactions—adopting positions that favour and facilitate movement.

For the 30 min session, the exercise program consisted of the following DD, DL, and DV positions; sitting; on all fours; kneeling; standing. Assistive devices used include the therapy table, mattress, Bobath ball, inflatable cylinder, balance disc, Sveltus, inflatable balance disc, trellis, and walking belt for recovery.

***Vojta and Bobath group***—the session lasted 40 min; it was carried out as follows: in the first 20 min, Bobath exercises were performed, followed by 15 min of Vojta stimulation with a 5 min break between the two therapies.

The therapy sessions were carried out in an outpatient office according to established appointment times, with a frequency of three times a week; during the rest of the days, the members were taught to perform light exercises and stimulating positions at home in order to complete the therapy carried out by the physical therapist. The therapy was administered over the course of seven months. Evaluations were performed once a month and compared against the motor development grid, with the patient being considered recovered when the chronological age corresponded to the motor development age.

***a.***  ***Vojta therapy*** [28,29]

Vojta therapy stimulates the brain to reflexively activate the two-movement complex-es in which all the components of locomotion are contained: “reflex crawling, reflex rolling”. At the body level, stimulation is carried out in 4 main areas on the extremities—the medial humeral epicondyle (EMH), lateral calcaneal tuberosity (TLC), radial styloid process (ASR), and medial femoral epicondyle (EMF)—and 5 secondary areas on the trunk—the medial edge of the scapula in the lower 1/3 (MMS), Acromion (A), VII-VIII intercostal space (SI), gluteal area (ZF), and anterosuperior iliac spine (SIA) [28].

The Vojta therapy improved gross motor function and dynamic locomotion and im-proved spatial-temporal parameters in children with spastic diplegia [2]. Early intervention by Vojta stimulation impacts the quality of neurological reflexes by modulating spontaneous motor abilities and postural responses [10,30,31,32]. Lim H. et al. demonstrated in a study carried out over a period of 2.8 years that Vojta therapy is more beneficial in hypertonic infants and can significantly improve posture and movement [33]. Vojta therapy can also be used as a treatment method for improving sitting position and diaphragmatic ascension during the breath in children with spastic cerebral palsy. This was demonstrated by Ha S.Y. et al. in 2018 [34].

**1.**  **Reflex rolling** 

**Figure 1:**
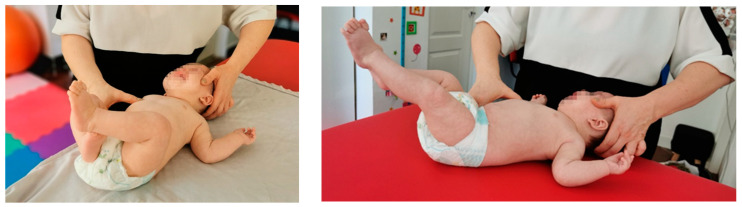
Stage 1 reflex rolling performed in Vojta therapy.

**Stage I:** The patient is positioned in the DD position, the upper and lower limbs are extended, and the head is directed towards the therapist, with its turning being inhibited by the resistance given on the zygomatic bone, the nuchal line, and the mastoid tuberosity. Stimulation of the chest area for 10–15 s triggers kinesiological reactions (Figure 1). 

It stimulates both the left and the right side of the child. These activations are repeat-ed 3 times for each part. 

**Stage II:** The patient is positioned in the DL, with the upper limb below and the greater trochanter supporting the body. The stimulation areas are also the CIA, and the stimulation duration is 10–15 s. Kinesiological response is obtained as follows: in the lower upper limb: shoulder blade—adhesion to the ribcage, scapulohumeral—90° flexion with external rotation, elbow—slight flexion and pronation, fist—dorsal extension and radial inclination, metacarpals—abducted with finger extension; upper limb: scapula—attachment to the ribcage, scapulohumeral—flexion, abduction, and external rotation, elbow—slight flexion with supination, fist—dorsal extension with radial inclination, metacarpals—abduction and extension of the fingers; lower hip—external rotation, slight flexion with a tendency towards extension, knees—flexion with a tendency towards extension, talocrural joint—inversion with supination, metatarsals—abduction and flexion of the fingers; upper hip: 90° flexion with abduction and external rotation, kneeling—90° flexion, talocrural joint—in medial position, metatarsals—abducted with fingers in medial position. Stimulation is provided for both the left and the right side of the child, these activations are repeated 3 times for each side.

**Stage III:** The patient is positioned in the DD, and the area of stimulation is MMS and EMF of the upper part. Stimulations lasts for 10–15 s, and the kinesiological response as specified for Stage II. Stimulation is provided for both the left and the right side of the child; these activations are repeated 3 times for each side.

**Stage IV:** The patient is positioned in the DD, and the area of stimulation is the MMS and the lower lateral femoral epicondyle. Stimulations lasts for 10–15 s, and the kinesiological response is as specified for Stage II. Stimulation is provided for both the left and the right side of the child; these activations are repeated 3 times for each side.

**2.**  **Reflex crawling**

This represents a complex of movements that contain all the essential components of displacement, representing the basic models of locomotion, thus ensuring postural coordination, a righting against gravity, as well as stepping movements of the upper and lower limbs.

The patient is positioned in the DV, the head rotated with the occipital part towards the therapist and the facial part towards the elbow pocket of the facial upper limb. The movement is carried out in a crossed pattern, where the right lower limb and the left upper limb move concomitantly and in the opposite direction; thus, the lower limb and the opposite upper limb support the body and move the trunk forward. By stimulating the areas, the activation of the entire body musculature is amplified, initializing the verticalization process. The upper limb on the occipital part is stretched loosely next to the trunk, and the lower facial limb is stretched loosely: the upper limb on the facial side—flexion 125°–130°, adduction, and external rotation; the elbow—flexion of 45° and pronation; the styloid apophysis—in the same line as the hip and facial shoulder, parallel to the vertebral column.

The first position applies to infants starting from the 8th month; it has no locomotion elements but is used for uprighting. We can use all the activation zones from the reflex crawl as well as the resistance points at the level of the head. The working position is at the edge of the therapy table; the patient is seated in a squatting position, the pelvis with the ischial tuberosity is resting on the heels, the feet are at the edge of the bed, out, knees are flexed at the level of the axilla with the calves parallel, the head is down and rotated to one side at 30°, the facial side is facing the facial arm, the facial arm is in trunk extension in the scapulo-humeral joint, extension 120°–135°, with support of the elbow (45°).

**Figure 2:**
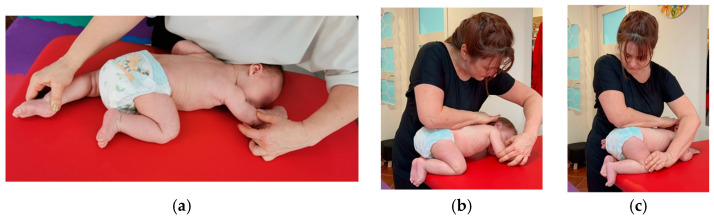
Reflex crawling performed in Vojta therapy: (**a**) stimulation in “two points”; (**b**,**c**) stimulation in “three points”.

**A.**  **Stimulation in “two points”** of support on the facial side is the EMH area and on the occipital side is the distal part of the tibia. The epicondyle area is stimulated for 10–15 s until obtaining the kinesiological response of the lower limb on the occipital side, which achieves reverse supination and inversion of the abducted metatarsals with flexion of the fingers, and for the lower limb on the facial side, an eversion position with the abducted metatarsals and finger extension (Figure 2a).
**B.**  **Stimulation in “three points”** of support on the facial side is the EMH area and on the occipital side is the distal part of the tibia; the proximal part of the tibia on the facial side is a kinesiological response, and the lower limb on the occipital side. The flexion of the fingers is accentuated, and on the lower limb facial part, a median position is obtained in terms of flexion and extension—respectively, inversion and eversion. These activations are repeated 3 times on each side (Figure 2b,c).

***b.***  ***Bobath therapy*** 

The fundamental principles of Bobath therapy are the suppression or elimination of the activity of pathological reflexes that help to reduce and normalize muscle tone and achieve postural control through rehabilitation procedures that are subjected to a gradual increase in number, intensity, and duration, thus preventing further complications such as contractures and deformations [35,36].

The Bobath therapy was aimed at improving automatic postural reactions, lifting re-actions, balance, and adaptive changes in muscle tone. The exercises proposed and ap-plied followed the level and the functional deficit at the start of the kinetic treatment.

**Figure 3:**
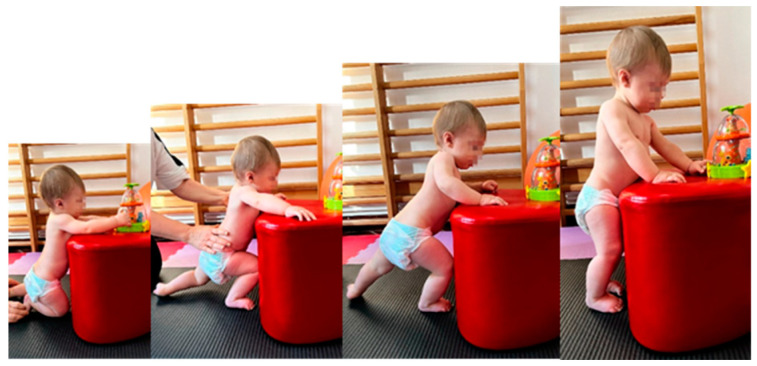
“Servant Knight” position.

1. Inhibitory reflex positions, in general, are partially opposite to the infant’s abnormal posture. First, the head and neck are positioned, then the trunk, shoulders, and hips, to achieve a redistribution of muscle tone as close to normal as possible.
  - By positioning the head, we activate the tonic reflexes of the neck to favour the flexion or extension of the upper or lower limbs.
  - To activate asymmetric cervical tonic reflexes, the head is positioned on the side of the interested limb, obtaining relaxation of the flexor tone; alternatively, we can mobilize it more easily by turning it to the opposite side.
  - To activate the tonic labyrinthine reflex, the infant is positioned in the supine position (DD), and by anterior flexion of the head and neck, with the positioning of the upper limbs crossed on the chest, a relaxation of the lower limbs is obtained; thus, their movement becomes freer, without a spastic contraction.
  - In the case of the opisthotonos position, to relax the extensor muscles of the neck, trunk, and limbs, the foetal position is adopted, with slight antero-posterior swings.
  - Infants who showed a tendency to crouch were held by the palms and lifted, obtaining extension of the head and limbs, in a reflex inhibitory position, facilitating easy movement of the limbs. The same relaxation can be obtained from the ventral decubitus (DV) position, lifting the infant’s head off the bed with one hand and holding the abdomen with the other hand, ensuring stimulation of the Landau reflex.
2. Exercise complexes are used to stimulate, by all means, the body’s equilibrium reactions, challenging and strengthening them through repetition.
  - From the sitting position, from the knees, and from all fours, small and short pressures are applied to the infant’s shoulder, pushing it in all directions, thus being taught to react by raising the arm on the side towards which the infant is being pushed. It is performed in two sets of five repetitions, with a 1 min break between sets.
  - For sitting up from DD, the return to the lateral decubitus is initiated, with support, on the body, on the forearm, then on the palm; the position is supported by the physiotherapist, with a socket at the level of the lower limbs. It is performed in two sets of five repetitions, with a 1 min break between sets.
  - The transfer from sitting to all fours is performed by loading of the upper limbs, using left or right lateral movement using a downward inclined plane. It is performed in two sets of five repetitions, with a 1 min break between sets.
  - Lifting in the quadrupedal position: from the DV, loaded support is performed on the upper limbs, gradually moving to the easy lifting of the pelvis by flexing the coxo-femoral joints, favouring support of the knees. Execution: 5 repetitions on the mat or with the help of the Bobath ball.
  - Initiating the verticalization by performing the “Servant Knight” position: the initial position is with support of both knees and support at the level of the upper limbs, by pulling up a lower limb that is bent (triple flexion—in the hip, knee, and foot) and pushing for verticalization, it is reached with support of both lower limbs to stabilize and balance the position. Execution: 2 sets of 5 repetitions on each lower limb, with a 1 min break between sets (Figure 3).
  - In the orthostatism position, using the inflatable disc, balance is stimulated; as an indispensable reaction to walking, the infant is easily unbalanced by antero-posterior and lateral pushing manoeuvres.

### 2.4. Statistical Analysis

By using GraphPad version 9 for quantitative data, we determined mean values and standard deviation (SD). Associations between a dependent variable (applied therapy method) and a series of independent variables (neurological diagnosis, ante-, peri-, and postnatal factors) was identified for logistic regression. Logistic regression was applied to determine if, by combining the two therapies in the case of patients with motor development disorders, the recovery of the motor deficit is achieved in a shorter period. Cox regression was applied to identify the recovery time for the combined method compared to the two methods used separately. 

For all the tests, the significance threshold was set to 0.05.

## 3. Results

The demographic characteristics of the study groups are represented in Table 1. The majority, 84 (77.7%) of the subjects, were from urban environments, and 24 (22.3%) were from rural areas. The median age of patients was 4 (1–6). The mean weight of the children included in this study was 3123 g (SD = 700). Children who benefited from the combined treatment had the highest average birth weight of 3241 g (SD = 654.47). The highest average age of the patients was found in those who were treated with the Vojta therapy (3.83 months, SD = 1.50). Most of the patients included in our study had an increase in APGAR greater than 8.

**Figure 4:**
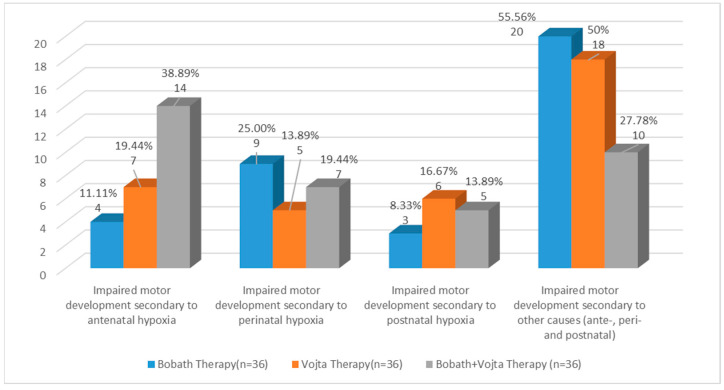
Classification of study groups according to the cause of the impaired development.

All the subjects included in the study had impaired motor development. Sixty infants (55.6%) had ante-, peri-, or postnatal hypoxia, and 48 infants (44.4%) suffered from other ante-, peri-, or postnatal causes. Data are shown in Figure 4.

**Figure 5:**
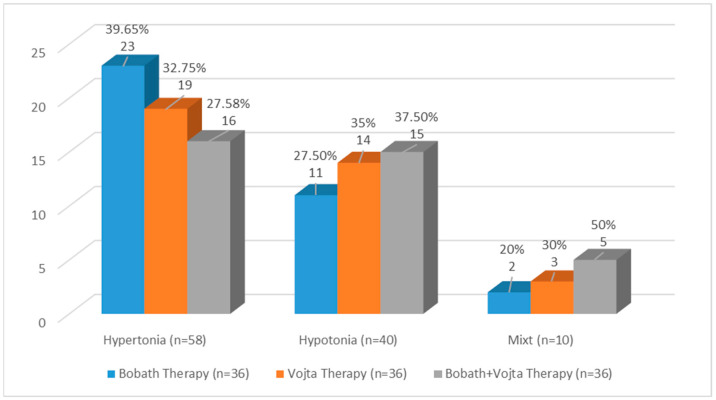
Classification of group therapy according to muscular tone.

Out of the study population, the most frequent muscular involvement was hypertonia, with 58 (53.7%) cases. The distribution of muscular involvement according to the therapy group is presented in Figure 5. Most of the infants presenting with hypertonia, 23 (39.65%), were treated with Bobath therapy, 15 (37.5%) of the infants presenting with hypotonia were treated with Vojta therapy, and 5 (50%) of the patients with a mixed muscle involvement were treated with the combined Bobath and Vojta therapy.

The characteristics of the study population based on the aetiology of ante-, peri-, or postnatal factors and the method used are detailed in Table 2.

In the first Bobath therapy group, the recovery for the 36 patients was performed over a period of 7 months. Twelve patients (33.33%) recovered in the fifth month of therapy. In the Vojta group, the recovery for the 36 patients happened over a period of 7 months. Eleven patients (30.56%) recovered in the fifth month of therapy. In the combined group, Bobath + Vojta, the recovery happened over six months, with 18 (50%) patients recovering after four months of therapy. Data are shown in Table 3. Furthermore, we noted the infant recovery according to the muscle tone, with data described in Table 4.

Based on the recovery time, a statistical significance was noted when observing the association of Bobath and Vojta therapies compared to Bobath alone (*p* = 0.0002; CI 95% 2.43–18.23) and Vojta alone (*p* = 0.0001; CI 95% 0.05–0.38) (Table 5).

The mean recovery time for combined Bobath and Vojta therapy was 3.97 ± 0.77 months, 95% CI% (3.710–4.234) (Table 6).

This hypothesis was additionally demonstrated by combining mean recovery time in months for the combined Bobath and Vojta therapies vs. Bobath alone and vs. Vojta alone, all *p* < 0.05 (Table 7).

## 4. Discussion

Ungureanu et al., in a pilot study performed on 12 children with cerebral palsy, ana-lysed the functional evolution of the biomechanical parameters that characterise the equilibrium. The authors demonstrated that the approach to body alignment through the Vojta method, on the one hand, and the facilitative inhibitory postures/exercises promoted through the Bobath method on the other allows for achieving symmetry [29]. In our study, the sequence of application in the same therapy session was initiated with Bobath therapy by applying the neuro-motor postural scheme of positioning in the dorsal decubitus (DD), ventral decubitus (VD), and lateral decubitus (DL). The correct printing of the form for the recovery (from DD to LD and LD to LD) and rolling schemes (DD to VD and vice versa) was assessed, followed by the transition from supine to sitting on one side, raising from DV to quadrupeds, moving to quadrupeds, kneeling followed by raising to orthostatism with passing through the knight position, sidewalk, and independent walk. It is necessary to use these movements according to the age and developmental level of the infants. At the same time, the aim was to improve spatial-temporal perceptions through motor activities to stimulate prehension and prevent postural alignment disorders that may result from force imbalances between the agonist and antagonist muscles at the level of the spine and in the upper and lower limbs. The second part of the therapy session is followed by the Vojta method, where the working position from the VD for the “crawling reflex” and from the dorsal or lateral decubitus for the “rolling reflex” are addressed; the combination of stimulation areas and starting positions is carried out in multiple variants depending on the reaction sought by the stimulation.

Our study evaluated the efficiency of Vojta and Bobath therapies based on the complete recovery time criteria as well as the characteristic of muscle tone (hypotonia, hypertonia, and mixed). Thus, for infants with a neurological picture dominated by muscle hypotonia, complete recovery by Bobath therapy was achieved in 5 months in more than 70% of the cases. Moreover, full recovery was achieved with Vojta therapy at five months in more than 92% of the cases. Our results also indicate that a combination of both therapies gives the best results, as we registered full recovery at five months in 93% of the infants. These results are similar to a comparative study performed between 2008 and 2012 at the School of Therapy and Social Work, Fresenius University of Applied Sciences Frankfurt, Germany, where the authors assessed the efficiency of Vojta therapy compared to Bobath in posture alignment. The results showed that both methods are efficient in postural asymmetry rehabilitation [37]. Inamura et al. demonstrated a significant improvement with Vojta therapy in 60% of the subjects with moderate motor developmental delay and 45.5% in subjects with severe motor developmental delay [38]. Other research focused on the rehabilitation of a six-month-old child with Soto’s syndrome by Vojta therapy, where the initial evaluation revealed six abnormal asymmetric postural responses and abnormal primitive responses as well as a spontaneous motricity level corresponding to 3 months of development. The results of this particular study show that after eight months of therapy, only four abnormal asymmetric postural reactions were identified, and the spontaneous motricity corresponded to 7.5 months [39]. According to our study, the subjects from the Vojta group recovered at seven months, when the level of motor development coincided with the chronological age.

Regarding infants with muscular hypertonia, for more than 50% of the cases, Bobath therapy offered complete recovery in 5 months, while the Vojta therapy proved success for less than 35% of the cases. By combining Bobath and Vojta therapy, complete recovery in 100% of the cases at five months after initiation was achieved. Our findings indicate a difference in therapy choice when analysing the mean recovery times between Vojta and Bobath therapy in favour of the former. But by combining them, the mean recovery time was shorter. Contrary to our results, on a study conducted by Zanon et al. in 2019 that compared Bobath to Vojta therapy in children with cerebral palsy, the authors concluded that there were no differences between therapies [4].

In neuromotor impaired development with a mixed clinical picture (hypotonia and hypertonia), the best results were found when combining Bobath and Vojta therapies at 3 and 4 months of age. Overall, our study suggests that combining the Bobath and Vojta therapies for infant neurorehabilitation was superior to Vojta or Bobath alone when considering muscle tone. However, if we eliminate the muscle tone criterion, the differences are unnoticeable regarding the complete recovery time. With Vojta intervention, the four-month recovery time was only 2.78% higher compared to Bobath, and at five months, the recovery time favoured the latter. But at seven months, there were no differences between the two groups. 

The limitation of this study is the small sample size. Another limitation of the study is related to the age of the infants evaluated, taking also other parameters into account, before birth but also during birth (for example. gestational weight at birth and APGAR score). An important limitation is the involvement of the parents in carrying out the therapy at home, including the lack of monitoring of the therapy carried out by the parents; a continuation of the therapy at the patient’s home is a very important factor for a faster recovery.

## 5. Conclusions

This prospective study aimed to evaluate the recovery of motor deficits in infants with impaired motor development secondary to neurological causes by implementing a therapeutic programme according to Bobath and Vojta therapy, individually or in combination. The comparative evaluation carried out by analysing the data regarding the application of the Bobath and Vojta methods showed that combining these two therapies reduces the recovery time of the motor deficit as compared to application of a single therapy.

## Figures and Tables

**Table 1:** Demographic characteristics of the study population based on therapy group.

Variable	Bobath Therapy (*n* = 36)	Vojta Therapy (*n* = 36)	Bobath + Vojta Therapy (*n* = 36)	*p*
Male Female	19 (52.78%) 17 (47.22%)	22 (61.11%) 14 (38.89%)	23 (63.89%) 13 (36.11%)	0.337
Urban Rural	26 (72.22%) 10 (27.78%)	32 (88.89%) 4 (11.11%)	26 (72.22%) 10 (27.78%)	0.145
APGAR score <8 ≥8	6 (16.67%) 30 (83.33%)	8 (22.22%) 28 (77.78%)	7 (19.44%) 29 (80.56%)	0.837
Birth weight <3000 g ≥3000 g	19 (52.78%) 17 (47.22%)	13 (36.11%) 23 (63.89%)	12 (33.33%) 24 (66.67%)	0.192
Premature	1 (2.78%)	3 (8.33%)	4 (11.11%)	
Age mean (SD)	3.64 (1.42)	3.83 (1.50)	3.81 (1.47)	
Birth weight Mean (SD)	2898 (747.49)	3229 (658.22)	3241 (654.47)	

**Table 2:** Classification of study groups according to the aetiology.

Variable	Bobath Therapy (*n* = 36)	Vojta Therapy (*n* = 36)	Bobath + Vojta Therapy (*n* = 36)
Antenatal factors	18 (50.00%)	14 (38.89%)	10 (27.78%)
Perinatal factors	11 (30.55%)	6 (16.67%)	16 (44.44%)
Postnatal factors	7 (19.45%)	16 (44.44%)	10 (27.78%)

**Table 3:** Classification of recovery time according to the duration of therapy.

Variable	Bobath Therapy (*n* = 36)	Vojta Therapy (*n* = 36)	Bobath + Vojta Therapy (*n* = 36)
Recovery after three months	3 (8.33%)	3 (8.33%)	10 (27.77%)
Recovery after four months	8 (22.22%)	9 (25.00%)	18 (50.00%)
Recovery after five months	12 (33.33%)	11 (30.56%)	7 (19.44%)
Recovery after six months	9 (25.00%)	9 (25.00%)	1 (2.78%)
Recovery after seven months	4 (11.11%)	4 (11.11%)	-

**Table 4:** Classification of infant recovery according to muscle tone.

Recovery	Hypotonia *n* = 40			Hypertonia *n* = 58			Mixt *n* = 10		
	Bobath Therapy (*n* = 11)	Vojta Therapy (*n* = 14)	Bobath + Vojta Therapy (*n* = 15)	Bobath Therapy (*n* = 23)	Vojta Therapy (*n* = 19)	Bobath + Vojta Therapy (*n* = 16)	Bobath Therapy (*n* = 2)	Vojta Therapy (*n* = 3)	Bobath + Vojta Therapy (*n* = 5)
Three months	-	4 (28.57%)	3 (20%)	2 (8.69%)	-	4 (25%)	1 (50%)	-	3 (60%)
Four months	3 (27.27%)	5 (35.71%)	8 (53.53%)	5 (21.73%)	2 (10.52%)	8 (50%)	-	2 (66.6%)	2 (40%)
Five months	5 (45.45%)	5 (35.71%)	3 (20%)	6 (26.08%)	4 (21.05%)	4 (25%)	1 (50%)	1 (33.3%)	-
Six months	1 (9.09%)	-	1 (6.66%)	8 (34.78%)	9 (47.36%)	-	-	-	-
Seven months	2 (18.18%)	-	-	2 (8.69%)	4 (21.05%)	-	-	-	-

**Table 5:** Association between the parameters that contribute to patient recovery based on therapy.

	Bobat + Vojta Therapy vs. Bobat Therapy			Bobat + Vojta Therapy vs. Vojta Therapy		
	*p*	OR	95% CI	*p*	OR	95% CI
Antenatal factors	0.87	1.11	0.26–4.63	0.91	1.11	0.17–7.10
Perinatal factors	0.30	0.43	0.09–2.08	0.23	2.95	0.50–17.48
Hypertonia	0.33	2.00	0.48–8.30	0.30	0.47	0.11–1.99
Mixt	0.19	0.18	0.01–2.38	0.92	1.10	0.13–9.14
Recovery after 4 (months)	0.0002 *	6.66	2.43–18.23	0.0001 *	0.14	0.05–0.38

* Statistical significance.

**Table 6:** Mean recovery times between therapies.

	Bobath Therapy	Vojta Therapy	Bobath + Vojta Therapy
Mean	5.11	5.02	3.97
Std. Deviation	1.19	1.13	0.77
95% CI	4.70–5.51	4.64–5.41	3.71–4.23

**Table 7:** Comparison of the recovery times between combined Bobath and Vojta therapy and Bobath/Vojta alone.

	Bobath + Vojta Therapy vs. Bobath Therapy	Bobath + Vojta Therapy vs. Vojta Therapy
*p*	*p* < 0.0001	*p* < 0.0001
95% CI	0.66–1.61	0.59–1.51

## Data Availability

The datasets generated and analyzed during the current study are available from the corresponding author on reasonable request.
